# The unskilled-and-unaware problem and performance feedback in monotonous, easily accustomed, and repetitive work

**DOI:** 10.1038/s41598-025-88457-8

**Published:** 2025-02-04

**Authors:** Yasuhiro Nakamoto, Tomoharu Mori

**Affiliations:** 1 Faculty of Informatics, Kansai University, Takatsuki, Japan; 2 College of Comprehensive Psychology, Ritsumeikan University, Ibaraki, Japan

**Keywords:** Monotonous, easily accustomed, repetitive work, Unskilled-and-unaware problem, Performance feedback, Psychology, Human behaviour

## Abstract

The current research examined whether people predict their performance on monotonous, easily accustomed, and repetitive (MEAR) tasks accurately and confirm the effects of performance feedback on their MEAR work predictions. Considering typing on the keyboard as an MEAR task in modern society, 128 university students were asked to type a 12-digit number displayed on each monitor within 5 minutes, explaining that there would be 75 typing problems and participants would receive 20 yen per correct answer. Additionally, participants were informed that they could earn money by reaching a self-determined performance target, with the condition that if they did not reach their self-set goal, they would not receive any earnings. The main findings were as follows: Regardless of the performance levels of typing on keyboards, only 23% of the participants reached the goal, indicating that the absence of performance feedback led to individuals’ overestimation of performance. In contrast, providing feedback significantly improved performance predictions: 76% of the participants reached the goal with feedback on their own performance, 70% with feedback on others’ performance, and 88% with feedback on both their own and others’ performance information. Specifically, the effects between one’s own and others’ performance feedback did not vary at statistically significant levels.

## Introduction

The unskilled-and-unaware problem (UUP) identified by Kruger and Dunning^1^, an influential study in this field, has shown that the unskilled lack the necessary metacognitive skills for accurate self-assessment predictions; hence, they would overestimate their actual performance. Alternatively, the skilled would predict theirs more accurately, an asymmetric error of performance predictions in metacognitive skill (see Refs.^2,3^ for reviews). Their argument for generating the UUP is that the unskilled have more difficulty recognizing their exact ability level than the skilled. Specifically, when the unskilled perceive that a task is easy, they tend to strongly overestimate their performances (e.g., see Ref.^4^). To reduce the overestimation of performance, specifically by the unskilled, numerous researchers (see Refs.^5–7^ for reviews) have focused on the role of performance feedback. Importantly, when examining the effects of performance feedback, some researchers (Refs.^4,8^, Studies 1 and 2; Refs.^9–12^, Task 2 of Study 2; Ref.^13^) employed quizzes such as general knowledge questions. In other research (Ref.^12^, Study 1; Refs.^14,15^), undergraduate students who participated were asked to predict the scores of the examination in the class (see Table 1).

**Table 1:** Previous studies.

Study	Task	Monetary incentives	Subjects	Feedback
Burson, Larrick, and Klayman^4^				
Study 1	Quiz	Only show-up fee	University students	No
Study 2	Quiz	Only show-up fee	University students	No
Study 3	“Word prospector” game	Only show-up fee	University students	No
Arkes, Christensen, Lai, and Blumer^8^				
Experiment 1	Quiz	No	17-33 aged subjects	Yes
Experiment 2	Quiz	No	17-37 aged subjects	Yes
Lichtenstein and Fischhoff^10^				
Experiment 1	Quiz	Hourly payment	University students	Yes
Experiment 2	Quiz	Hourly payment	University students	Yes
Hacker, Bol, Horgan, and Rakow^14^				
Score in exams		No	University students	Yes
Stone and Opel^13^				
Quiz (art history)		Yes	University students	Yes
Moore and Cain^11^				
Experiment 1	Quiz	Yes	University students	Yes
Experiment 2	Quiz	Yes	University students	Yes
Grossman and Owens^9^				
Quiz		Yes	University students and staffs	Noisy
Ryvkin, Krajc, and Ortmann^12^				
Study 1	Score in exams	Yes	University students	Yes
Task 1 in study 2	Math	Yes	University students	Yes
Task 2 in study 2	Quiz	Yes	University students	Yes
Sabater-Grande, Nikolaos Georgantzís and Herranz-Zarzoso^15^				
Experiment	Score in exams	Yes	University students	Yes

Unlike the tasks in the abovementioned studies, the current research focused on monotonous, easily accustomed, and repetitive (MEAR) work, which is represented by sorting, packaging, inspection jobs in the manufacturing industry, data entry business in the service industry, and everyday activities such as cutting and washing while cooking and typing on the physical keyboard. Notably, MEAR work plays an important role in creating employment opportunities and reducing unemployment, particularly for lower-skilled workers and people living in developing countries, which is an engine that supports economic growth. Thus, recognizing the skills of workers in MEAR work is important for opening up opportunities for advancement and leading to higher quality work. However, in this field, insufficient attention has been devoted to MEAR work. To our knowledge, this research was the first to focus on the UUP in MEAR work and to examine how accurately people make predictions of MEAR work performance. Based on the UUP, it could be expected that the unskilled would not make accurate self-assessment predictions, while the skilled would succeed. However, for MEAR tasks, there may not be a significant difference in performance between the unskilled and the skilled. If this is the case, the UUP would not be observed, and both groups would either be able to make accurate self-assessment predictions or both would be inaccurate. Furthermore, the current research investigated the effects of performance feedback on their predictions of MEAR work.

The current research regards typing on the keyboard as a typical MEAR task. This is because many people in modern societies, including the participants (undergraduate students in a Japanese university), frequently type on on the keyboard and consider the work monotonous and repetitive. For instance, the institution from which participants were recruited for the current research reported that almost all students had the opportunities to use the computers frequently. In detail, 76.2% of students had their own computer and 14.2% used the parent’s computer in their own home. Moreover, the university has many personal computers in each building, and the students can use them freely. A laboratory experiment was conducted to test participants’ performance predictions related to typing on the keyboard. Before performing the typing task, participants were asked to predict how well they could type. Thereafter, it was tested whether the prediction was correct and what factors influenced the predictions.

There are two main differences between existing studies and the current research. The first difference is the uncertainty of the next task. As quizzes and examination problems cover various kinds of content, the subsequent quiz and examination problems are unpredictable. Alternatively, the next task in MEAR work is predictable owing to the monotonous repetition. Therefore, people are able to predict their MEAR work performance more clearly compared to that in quizzes and exams. Moreover, as mentioned in Arkes et al.^8^, because a “trickery” hypothesis exists owing to the uncertainty of the quiz that comes up, people may be skeptical about whether they have been tricked. However, MEAR work involves little uncertainty and is not considered tricky. The second difference is the introduction of monetary incentives, which encourage people to make more accurate predictions (see Ref.^16^, for a review and Ref.^12^, Section 1.2). In this research, monetary incentives were provided to encourage participants to predict their performance more accurately. All participants applied for the experiment to obtain earnings. Moreover, all participants were asked to set a performance target of typing on the keyboard in the test task before implementing it. They were informed that they would obtain the targeted earnings on attaining the performance target; otherwise, they would receive nothing. In other words, participants wanted to increase their targeted earnings by setting higher goals; however, they recognized that it would increase the possibility of no earnings due to the non-achievement of self-set goals. Conversely, the monetary incentives underlying accurate predictions in the existing studies may be weak (see Table 1 for the reviews of related literature). Arkes et al.^8^, Burson et al.^4^, Lichtenstein and Fischhoff^10^, and Hacker et al.^14^ did not offer any incentive to provide accurate performance estimates. Additionally, participants in the works of Stone and Open^13^, Ryvkin, Krajč, and Ortmann^12^, and Sabater-Grande et al.^15^ were motivated by monetary incentives. However, unlike the current research, as their experiments were conducted during class sessions, all participants were not recruited for the experiments.

## Materials and methods

Our experiment participants, recruited from the pool of subjects maintained by the Center for Experimental Economics at Kansai University in Japan, were undergraduate students. There were 128 participants, including 59 men and 69 women (each session consisted of approximately 15 participants). Our experiments were conducted at the experimental economics laboratory in the university. Each session lasted 30 minutes on average.

Upon arrival, participants were seated in isolated cubicles. The instructions were read aloud, explaining that participants would obtain a 1,000-Japanese-yen show-up fee (approximately 10 US dollars). Each participant was informed about the purpose of the research, personal data protection, the procedure for withdrawing from the experiment, the compensation for participation, and the duration of the experiment. Afterward, they signed a consent form. Then, all participants were informed that their task would be to type the 12-digit number displayed on each monitor within 5 minutes, referred to as the typing task in this study. Specifically, as mentioned by Giannouli^17,18^, performance in MEAR work for extended time may be influenced by perseveration. Thus, in the experiment, typing time on the keyboard was limited to 5 minutes to prevent perseveration from complicating self-assessment predictions.

Participants reviewed samples of the randomly arranged 12-digit number on each monitor, such as “392832944950” and “958375930201.” In addition, to further limit the requisite skills, participants were allowed to choose a familiar typing tool: either the QWERTY keyboard or the numeric keypad (10 keys) adjacent to it. Next, we explained that there would be a total of 75 typing problems and that participants would receive 20 yen per correct answer; therefore, the maximum earnings would be 1,500 yen (=20 yen $\times$ 75 problems). In addition, participants were informed that they could not return to the previous problem after moving on to the following problem. They were also informed about the role of goal setting, which allowed them to obtain earnings by reaching a self-determined performance target. Participants were told that if the number of correct answers exceeded the self-set goal of correct answers, they could obtain “20 yen $\times$ the number of the self-set goal.” In contrast, if participants did not reach the self-set goal, they would not receive any earnings. Thus, participants were motivated to set higher goals; however, higher goals could decrease the probability of reaching those goals. Furthermore, a concrete explanation was provided using the following example: Suppose that a participant sets 20 as the self-set goal; the obtained earnings would be 400 yen (=20 yen $\times$ 20 problems) if they achieved the self-set goal. Notably, even if the number of correct answers exceed 20, such as 25 and 75, the earnings do not change and amount to 400 yen based on the self-set goal. Thereafter, they were asked to reveal the targeted number of correct answers in the typing task. After completing the test task, participants were asked to answer the post-experimental questionnaire.

To determine the sample size for this research, a power analysis was conducted. This research aimed to detect a large effect size (Cohen’s *d* = 0.8) with a significance level ($\alpha$) of 0.05 and a desired power (1-$\beta$) of 0.8 for pairwise comparisons. Based on these parameters, the power analysis indicated that a minimum of 26 participants per group was required to achieve sufficient statistical power. To account for potential dropout, the sample size was increased by 30$\%$. Then, four groups were created: the Control ($n =$ 31 of the total 128 participants), Exp(erience) ($n =$ 34), Info(rmation) ($n =$ 30), and Info(rmation)Exp(perience) ($n =$ 33) groups, as shown in Table 2. None of the four groups had any participants who dropped out. The variables, except for Age and Male, are explained later. The difference in the number of participants between the groups was caused by the difference in dropout rates. Groups were determined by the experimental session, which facilitated scheduling and logistical management.

**Table 2:** Summary statistics.

	Control (N=31)		Exp (N=34)		Info (N=30)		InfoExp (N=33)	
	Mean	Std. dev.	Mean	Std. dev.	Mean	Std. dev.	Mean	Std. dev.
Male	0.42	0.50	0.47	0.51	0.47	0.51	0.48	0.51
Age	20.45	1.69	20.56	1.52	20.50	1.11	20.88	1.22
Job	0.84	0.37	0.76	0.43	0.93	0.25	0.85	0.36
Math	0.10	0.30	0.06	0.24	0.20	0.41	0.09	0.29
Loss propensity	2.77	1.45	3.12	1.65	3.43	1.59	3.50	1.93
Extraversion	3.84	1.49	3.62	1.20	3.85	1.40	4.36	1.64
Agreeableness	4.95	1.03	4.68	1.27	4.72	1.72	5.27	1.13
Conscientiousness	3.24	1.26	2.97	1.07	3.12	1.42	3.45	1.35
Neuroticism	4.82	1.14	4.66	1.35	4.22	1.45	4.34	1.48
Openness	3.95	1.25	4.03	1.39	3.90	1.43	4.00	1.25

The p-values of the F-tests for the balance test are all greater than 0.1

**Fig. 1:**
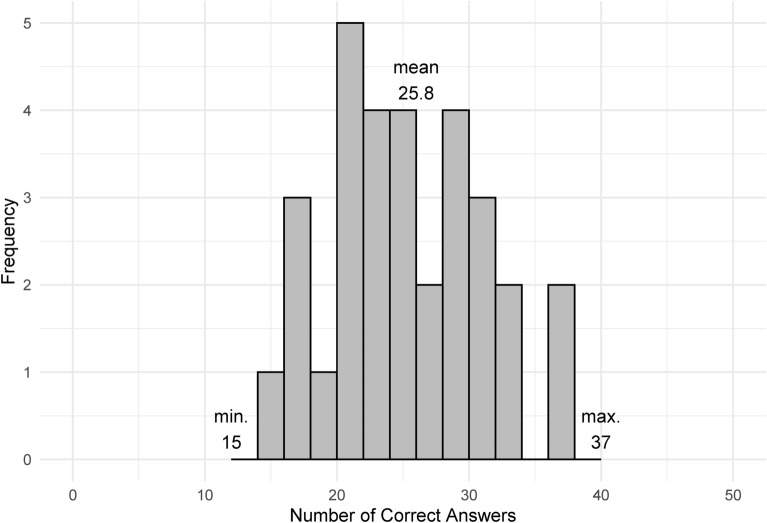
Number of correct answers in control group.

Considering MEAR’s work with monetary incentives, participants were randomly assigned to one of four groups: Control, Exp, Info, or InfoExp. The groups differed in how the performance feedback was introduced. Each group was constructed as follows: First, participants in the Control group did not receive any performance feedback. Therefore, they did not have any way to know their performance. Second, in the Exp group, participants were additionally informed that the practice task would be implemented before the test task, and they did not receive any earnings for the practice task. After participants in the Exp group typed within 5 minutes for the practice task where the same 12-digit numbers were not used for the practice and test tasks, they reviewed their performance (the number of correct answers) on the monitor. Consequently, participants in the Exp group were able to gauge their achieved typing performances in the experiment. Third, in the Info group, participants viewed the distribution of correct answers in the keyboard typing task from the other 30 participants before the test task (see Fig. 1). These 30 participants had been newly recruited in a satellite campus of the same university, while the experiment was conducted at the main campus. To demonstrate the reliability of the prior information shown in Fig. 1, following Luckett and Eggleton^6^, participants in the Info group were additionally informed about source attributes (i.e., the 30 participants in the prior investigation were the undergraduate students in the satellite campus of the same university). By looking at Fig. 1, they understood the mean, maximum, and minimum levels in addition to the distribution of the correct answer. For example, based on Fig. 1, they might have understood that it would be difficult to achieve correct answers for all 75 problems within 5 minutes; alternatively, they might have recognized that they would be able to answer at least a minimum of 15 problems. Primarily, participants in the Exp group did not confirm others’ performances in Fig. 1, whereas those in the Info group did not practice beforehand; hence, they did not recognize their own performance. Alternatively, participants in the InfoExp group practiced typing, similar to the Exp group, and viewed the distribution of the correct answer, as in the Info group, implying that they were able to recognize both others’ and their own performances.

Finally, after completing the test task, participants were asked to answer the post-experimental questionnaire by providing the following information: gender, job, mathematical disposition, personality traits, and degree of loss aversion. As for the job, participants were asked to answer whether they had a part-time job (= 1) or not (= 0), which is denoted by the variable “Job” in Table 2. For mathematical disposition, participants were asked whether mathematics was one of their favorite subjects, corresponding to the variable “Math” in Table 2. This is because participants whose favorite subject is not mathematics may have disliked the 12 randomly arranged numbers; therefore, their performances may have been poorer than those whose favorite subject is mathematics.

Next, considering that Kruger and Dunning^1^ have argued that a lack of metacognition by the unskilled is the main element that leads to the overestimation of performances, participants were asked to answer the Big Five personality test. This is because there is robust evidence that metacognitive beliefs are significantly correlated with the Big Five personality traits (e.g. Ref.^19^). For instance, when assessing the contribution of personality to metacognition on problematic Facebook use among young adults, Marino et al.^19^ showed that emotional stability influences metacognition (negative beliefs about thoughts and cognitive confidence). To measure personality traits (see Table 3), this research used the Japanese version of the 10-Item Personality Inventory^20,21^, which was a measure of the Big Five (extroversion, agreeableness, conscientiousness, neuroticism, and openness in Table 3) personality dimensions^22^. The ratings of the two related items were averaged, generating the score of the personality traits in each dimension. For instance, the score of extroversion was calculated based on scores of Questions A and F in Table 2, where the score given in Question F was arranged in the opposite direction because the content in Question A was opposite to that in Question F. Each dimension was rated with one positively and one negatively keyed item, and each item was scored on a 7-point scale ranging from 1 (strongly disagree) to 7 (strongly agree).

Using Table 4, participants were asked to reveal loss-averse propensities in a coin-toss game, informing them that they would not receive any earnings in this problem. It could be predicted that participants who disliked loss tended to set a lower number of correct answers as their own goal because they strongly sought to avoid losing all earnings. For instance, if participants selected B (A) in all problems, the degrees of loss aversion would be very large (very small). This research treated the switching timing as the data in the empirical analysis in Section 3; however, these data did not impact the self-set goals at statistically significant levels. The mean level and standard deviation are given by “Loss propensity” in Table 2.

**Table 3:** Big five.

	I see myself as;	Strongly	Moderately	A little	Nor disagree	A little	Moderately	Strongly
A	Extraverted, enthusiastic.	1	2	3	4	5	6	7
B	Critical, quarrelsome.	1	2	3	4	5	6	7
C	Dependable, self-disciplined.	1	2	3	4	5	6	7
D	Anxious, easily upset.	1	2	3	4	5	6	7
E	Open to new experiences, complex.	1	2	3	4	5	6	7
F	Reserved, quiet.	1	2	3	4	5	6	7
G	Sympathetic, warm.	1	2	3	4	5	6	7
H	Disorganized, careless.	1	2	3	4	5	6	7
I	Calm, emotionally stable.	1	2	3	4	5	6	7
J	Conventional, uncreative.	1	2	3	4	5	6	7

**Table 4:** Coin-toss game.

Number	Question	Chice A	Chice B
1	Heads: -100 YEN; Tails: +600 YEN	Participate	Not Participate
2	Heads: -200 YEN; Tails: +600 YEN	Participate	Not Participate
3	Heads: -300 YEN; Tails: +600 YEN	Participate	Not Participate
4	Heads: -400 YEN; Tails: +600 YEN	Participate	Not Participate
5	Heads: -500 YEN; Tails: +600 YEN	Participate	Not Participate
6	Heads: -600 YEN; Tails: +600 YEN	Participate	Not Participate
7	Heads: -700 YEN; Tails: +600 YEN	Participate	Not Participate

## Results and discussion

**Fig. 2:**
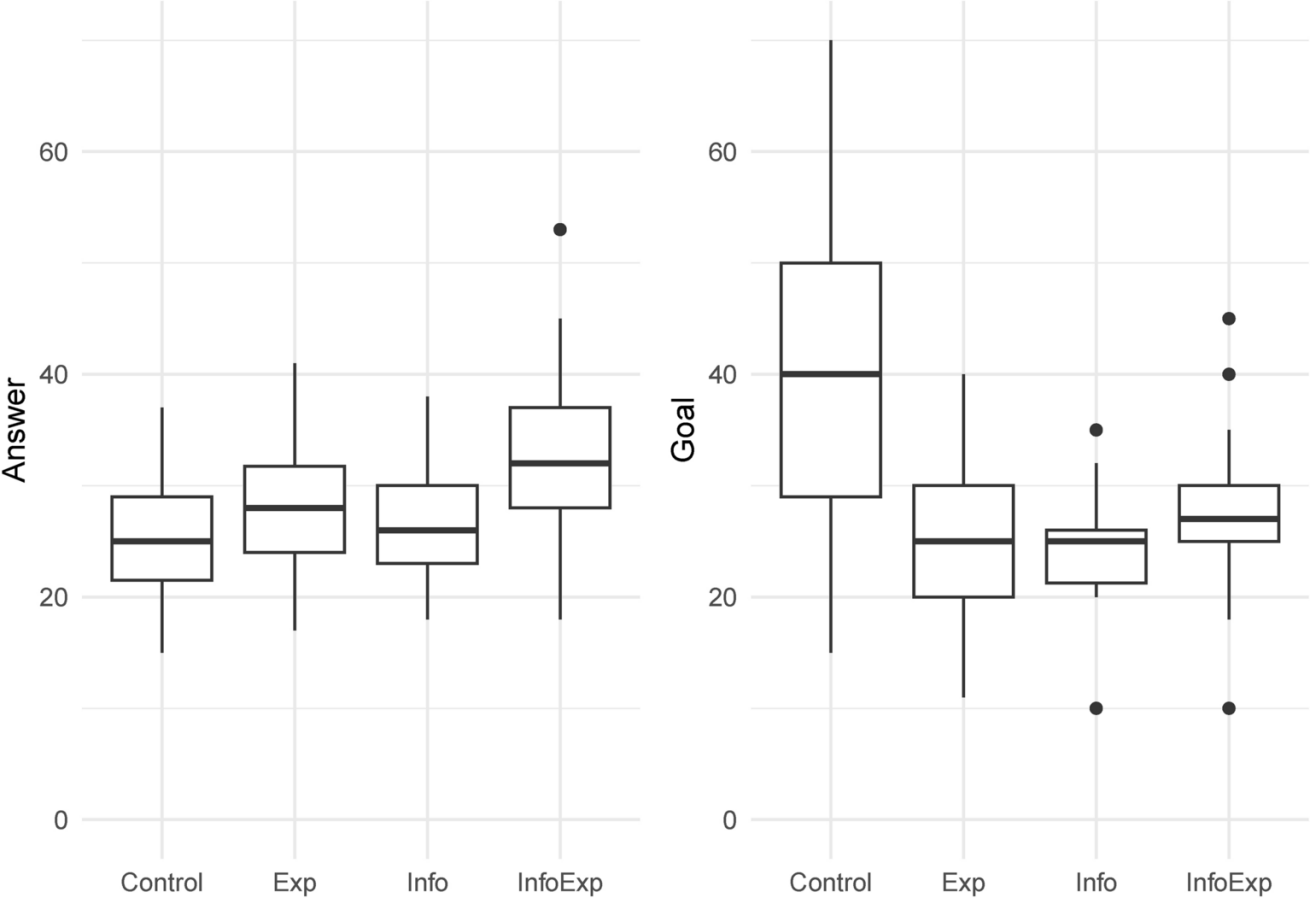
Boxplot of the number of answer and goal.

**Fig. 3:**
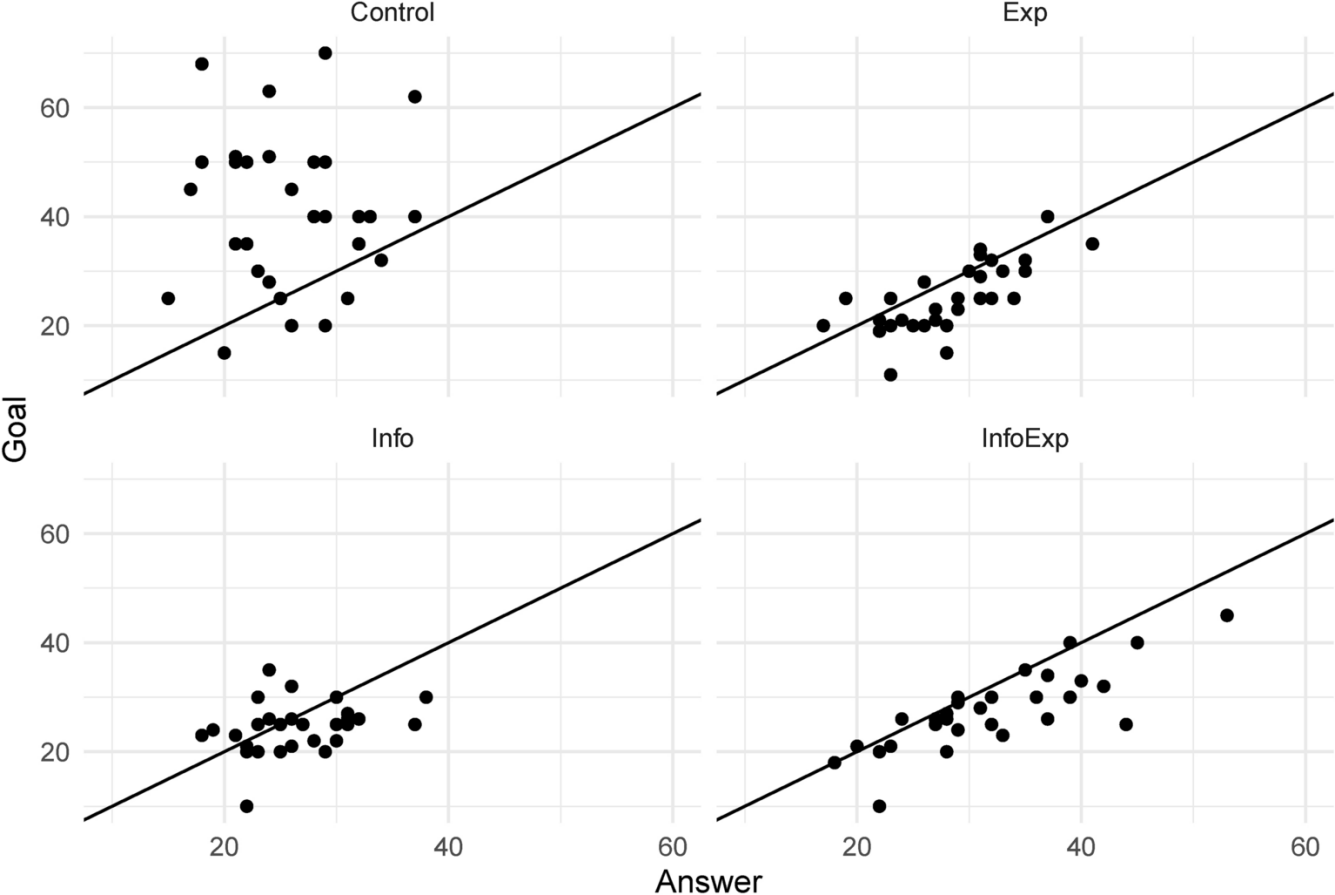
Scatterplots of answer and goal with 45 degree lines.

Table 5 presents a summary of the experimental results. The table shows the mean and standard deviation of the number of correct answers in each group (Answer), the goal set by participants (Goal), and the indicator variable, indicating whether the goal was reached in the number of correct answers (Success). Figure 2 shows box plots for Answer and Goal. Figure 3 presents the scatter plots for Answer and Goal, with 45-degree lines for each group. As the 45-degree line shows all points where the number of correct answers is equal to the self-set goal, the points to the upper left of the 45-degree line mean that the number of goals is greater than the performance; hence, the participants did not achieve the goal. Conversely, the points to the bottom right of the 45-degree line mean that the number of goals is less than the performance, implying that the participants achieved the goal. Table 6 shows the group differences for each variable. It also presents the $p-$values for the $t-$test for the difference of means, Wilcoxon rank-sum test, and the test of equal variances.

**Table 5:** Summary of the results.

	Control (N=31)		Exp (N=34)		Info (N=30)		InfoExp (N=33)	
	Mean	Std. dev.	Mean	Std. dev.	Mean	Std. dev.	Mean	Std. dev.
Answer	25.81	5.75	28.03	5.58	26.50	4.81	32.03	7.71
Goal	40.48	14.36	24.94	6.21	24.30	4.65	27.70	6.78
Success	0.23	0.43	0.76	0.43	0.70	0.47	0.88	0.33

Answer represents the number of correct answers. Goal represents the goal set by subjects themselves. Success takes the value one if Answer is equal to or greater than Goal, otherwise zero

**Table 6:** Pairwise comparisons for groups.

Comparison	Variable	Difference	Effect size	t-test	Ranksum	Variance	Exact
Exp - Control	Answer	2.223	0.393	0.119	0.124	0.868	-
Exp - Control	Goal	-15.543	-1.428	0.000	0.000	0.000	-
Exp - Control	Success	0.539	0.524	-	-	-	0.000
Info - Control	Answer	0.694	0.131	0.611	0.593	0.342	-
Info - Control	Goal	-16.184	-1.506	0.000	0.000	0.000	-
Info - Control	Success	0.474	0.458	-	-	-	0.000
InfoExp - Control	Answer	6.224	0.911	0.001	0.001	0.109	-
InfoExp - Control	Goal	-12.787	-1.150	0.000	0.000	0.000	-
InfoExp - Control	Success	0.653	0.646	-	-	-	0.000
Info - Exp	Answer	-1.529	-0.292	0.244	0.182	0.419	-
Info - Exp	Goal	-0.641	-0.116	0.639	0.968	0.117	-
Info - Exp	Success	-0.065	0.000	-	-	-	0.584
InfoExp - Exp	Answer	4.001	0.596	0.018	0.031	0.069	-
InfoExp - Exp	Goal	2.756	0.424	0.088	0.063	0.614	-
InfoExp - Exp	Success	0.114	0.084	-	-	-	0.340
InfoExp - Info	Answer	5.530	0.851	0.001	0.002	0.012	-
InfoExp - Info	Goal	3.397	0.579	0.023	0.018	0.043	-
InfoExp - Info	Success	0.179	0.180	-	-	-	0.120

Effect size is cohen’s d (for t-test) and phi (for fisher’s exact test). ‘t-test’ means Welch’s t-test. ‘Ranksum’ means Wilcoxson ranksum test. ‘Variance’ means F-test of equality of variances. ‘Exact’ means Fisher’s exact test

In the Control group, the mean Goal level was 40.48, the highest among all groups. Compared to the other groups, the results of the $t-$test and Wilcoxon rank-sum test indicated statistical significance. Alternatively, the mean Answer level in the Control group was 25.81, the lowest among all groups, with a significant difference only compared to the InfoExp group. These statistics implied that many participants do not achieve their goal. Success shows that only 23$\%$ of the participants in the Control group reached the goal, the lowest percentage among all groups. Figure 3 also showed that Goal exceeded Answer for many participants. Compared to the other groups, the results of the Fisher’s exact test indicated statistical significance.

Although all participants received information about the task’s properties, thereby reducing ambiguities regarding the task^23^, only the Control group exhibited extreme misjudgment regarding goal setting. Participants might have set extremely high goals, much above their performance levels, because of overconfidence regarding their typing on the keyboard, which might stem from the following two reasons. First, existing studies (e.g. Refs.^11,24,25^) have defined an easy task as one that people generally feel capable of doing, with relatively high absolute performance, and argued that people tend to overestimate their performance due to overconfidence. As typing on the keyboard as MEAR work was easy for the undergraduate students who participated in the experiment, participants tended to overestimate their performances.

Second, only participants in the Control group did not receive any feedback regarding task performance, unlike in the other groups. In other words, as those who participated in the Control group did not know about their own and others’ performances, they had no choice but to judge their performances through their past keyboard typing experiences. However, Kahneman & Tversky^26^ mentioned that when making an intuitive prediction, such as goal setting in the current research, people tend to disregard distributional information about the outcomes in similar cases, leading to a major error in intuitive prediction. That is, if the similar cases of the keyboard typing task conducted in the experiment corresponded to participants’ past experiences of keyboard typing, participants could develop an understanding of the distribution of their typing performance based on these past experiences (e.g., speed and accuracy). However, participants in the Control group would disregard the clues provided by their repeated past experiences when predicting their performance. If this were the case, the undergraduate students’ ease of keyboard typing may have generated overconfidence about the typing task, leading to an overestimation of expected performance.

In the Exp group, the mean value of Goal was 24.94, approximately 60$\%$ of that of the Control group. The standard deviation of Goal was reduced to less than half of that of the Control group. Tests of equal variances with the Control group indicated that the variances differed significantly. However, the mean value of Answer was 28.03, slightly higher than that of Goal. The value was higher than that for the Control group, but the difference was not significant. These results suggested that the success rate of the Exp group is as high as 76%, more than triple that of the Control group. These results indicated that experience increases the predictive accuracy of one’s performance, in terms of both mean and variance, as well as the probability of success.

The result of the Info group was similar to that of the Exp group. The mean value of Goal was 24.30, approximately 60$\%$ of that of the Control group. This value was smaller than the Control group mean of 25.8, as shown in the experiment. The standard deviation of Goal was reduced to less than one-third of that of the Control group. The mean value of Answer was 26.50, almost equivalent to that of Goal, with little difference compared to the Control group. The success rate in the Info group is 70$\%$. These values were close to their Exp group counterparts. None of the differences resulted in statistically significance. The results suggested that the same effect as in the practice task could be achieved by showing participants the distribution of correct answers in advance.

The results of the InfoExp group were highly different from those of the Control group and slightly different from the Exp and Info group results. The mean value of Goal was 27.70, which was significantly smaller than that of the Control group. It was significantly larger than that of the Info group and slightly larger than that of the Exp group; however, the latter difference was not statistically significant. The combined effect of experience and information may have facilitated higher goals. The mean value of Answer was 32.03, the highest among all groups; the difference between the Control and Info groups was significant, suggesting a difference compared to the Exp group. The success rate was also the highest, at 88$\%$, compared to the other groups. Figure 3 showed that Answer exceeded Goal for almost all participants in the InfoExp group and even those below the 45-degree line, where the difference between Answer and Goal was small.

These results suggested that performance feedback was crucial for personal goal setting irrespective of the source of feedback (individuals or others) in the current research. In addition, the effects of individuals’ performance feedback or joint effects of individual and others’ performance feedback generated improved performance.

The current research found that although MEAR work performance was more predictable than that in quiz and examination problems, and the overestimation did not result in any financial gain, many participants overestimated their performances without any feedback information in the Control group. Notably, not only poor but also high performers overestimated their performances, that is, there was no asymmetric prediction between the unskilled and skilled and no UUP was identified. Intuitively, as participants in the Control group had to judge their performances through experience, the easy task of typing on the keyboard led to overconfidence and a large divergence between the self-set goal and actual performance. Next, the performance feedback greatly reduced the overestimation of MEAR work performance. Specifically, considering the results in the Exp and Info groups, as there was no statistically significant difference in the effects between one’s own and others’ performance feedback, performance feedback, regardless of the kind, would work effectively. These findings are of relevance for a realistic society. For instance, when employers interview potential employees for MEAR work, the latter tend to overestimate their performances in the absence of performance feedback, regardless of actual performance levels. Alternatively, even if potential employees do not practice beforehand, information about others’ performance allows potential employees to predict their MEAR work performances accurately, which makes recruitment interviews more efficient.

Finally, Table 7 presents the results of the regression analysis of the variables analyzed so far on group dummies and individual characteristics. The odd columns denote models with only group dummies as independent variables, and the even columns represent models with individual characteristics added to the independent variables. The individual characteristics did not significantly influence the effect of group dummies in any of the analyses. The coefficients of the group dummies and adjusted coefficients of determination changed slightly after adding the independent variables. Considering the individual coefficients, only Agreeableness and Neuroticism had a suggestive negative impact on goal setting. The negative coefficient of Agreeableness suggested that sympathetic, warm, and cooperative participants lowered their self-set goals to set goals similar to those of others. The negative coefficient of Neuroticism implied that anxiety and worry about whether they can achieve their goals may adversely affect their goal-setting behavior because anxious and worried individuals tend to set more attainable goals to avoid potential failure or disappointment.

**Table 7:** Regressions.

	Answer		Goal		Success	
	(1)	(2)	(3)	(4)	(5)	(6)
Constant	25.806	19.895	40.484	56.094	0.226	$-$0.436
	(1.032)	(7.769)	(2.580)	(13.816)	(0.076)	(0.768)
Exp	2.223	2.269	$-$15.543	$-$15.730	0.539	0.552
	(1.408)	(1.489)	(2.791)	(2.752)	(0.106)	(0.110)
Info	0.694	0.361	$-$16.184	$-$17.814	0.474	0.508
	(1.355)	(1.501)	(2.716)	(2.727)	(0.114)	(0.121)
InfoExp	6.224	6.322	$-$12.787	$-$12.991	0.653	0.660
	(1.693)	(1.795)	(2.837)	(2.827)	(0.096)	(0.109)
Male		$-$1.983		$-$1.896		$-$0.057
		(1.216)		(1.708)		(0.088)
Age		0.360		$-$0.344		0.032
		(0.342)		(0.681)		(0.036)
Job		$-$0.304		$-$0.094		$-$0.029
		(1.204)		(2.341)		(0.124)
Math		1.159		4.831		0.034
		(2.028)		(2.754)		(0.131)
Loss propensity		0.124		0.239		$-$0.042
		(0.453)		(0.557)		(0.028)
Extraversion		0.008		0.563		0.009
		(0.443)		(0.518)		(0.024)
Agreeableness		$-$0.377		$-$1.190		0.009
		(0.410)		(0.515)		(0.031)
Conscientiousness		0.679		0.114		$-$0.012
		(0.454)		(0.742)		(0.034)
Neuroticism		$-$0.399		$-$1.374		0.007
		(0.430)		(0.525)		(0.033)
Openness		0.184		0.294		0.024
		(0.448)		(0.626)		(0.036)
Num. obs.	128	126	128	126	128	126
R2	0.140	0.215	0.359	0.436	0.268	0.305

Robust standard errors are in parentheses. Two samples are ommited due to missing values

## Conclusion

Considering typing on the keyboard as a MEAR task in modern society, the current research re-investigated the effects of feedback on the predictions of one’s own MEAR work performance. The main findings are presented as follows: First, irrespective of the actual performance levels, many participants overestimated their performance when no performance feedback was provided. Second, even if participants did not practice, the feedback regarding others’ performances greatly improved their predictions, and its effect was the same as that of the feedback regarding their own performance.

The effects of feedback information on overestimation can potentially be applied to various tests and tasks beyond MEAR tasks, such as intelligence quotient (IQ) and emotional quotient (EQ) tests. Recent research^27^ has reported that younger males overestimate their IQ and EQ compared to younger females, while older females overestimate theirs compared to older males. The current research suggests that providing this research information to participants as preliminary information could significantly change the results.

## Supplementary Information

Supplementary Information.

## Data Availability

The dataset in this study is available from the corresponding author upon reasonable request.

## Declarations

### Accordance statement

All methods were performed in accordance with the relevant guidelines and regulations.
