# Supplementary material for: The unskilled-and-unaware problem and performance feedback in monotonous, easily accustomed, and repetitive work
Source: Sci Rep. 2025 Feb 4;15:4222. doi: 10.1038/s41598-025-88457-8 (PMC11794710; doi:10.1038/s41598-025-88457-8)
Supplement: Supplementary file 1 — Supplementary Information. [file 41598_2025_88457_MOESM1_ESM.pdf]

Experiment

memo: This is the control group.

Please complete the following forms

- Please read the entire Economic Experiment Explanatory Document.

- Then, please fill out the following:
  - Economic Experiment Consent Form
  - Request for Bank Transfer Form

Economic Experiment Consent Form

- This section explains the important aspects of the Economic Experiment Consent Form.

- The experiment will be a typing task. It is scheduled to take 30 minutes, including payment.

- Your decisions and answers are guaranteed to be anonymous.

- If you are not satisfied with the specifics of the experiment, you can withdraw from participation at any time after reading the explanation. In such case, you will receive remuneration for your visit to the laboratory.
  - Payment is by bank transfer.
- If you have any questions, please ask the person in charge of the experiment.
- We will collect the Experiment Consent Form and Bank Transfer Request Form.

Payment of Participation Fee

- Thank you very much for your participation.
- The participation fee of 1,000 yen will be paid first.

Task

Rules of the show 1

- You will type the 12-digit number displayed on your monitor correctly.
- One problem is at 20 yen per correct answer.
- There will be a total of 75 typing problems (=20 YEN ×75 problems=1500 YEN (MAX)).
- You have 5 minutes.
- All problems consists of the 12-digit number (below)
- You can use the QWERTY keyboard or thenumeric keypad (10 keys) next to the QWERTY keyboards.

Exmples of typing problems

392832944950

958375930201

094726184959

Rule of the show 2

- You will be asked to set your target number of correct answers.
  - If you answer as many or more questions correctly than your target number of correct answers, you will be paid an amount equal to your target number of correct answers.
  - If you do not reach your target number of correct answers, you will only receive the participation fee.
- One problem is at 20 yen per correct answer.
- For example, let's say that your target number of correct answers is 10.
  - If you get more than 10 correct answers, you will get an additional 200 yen. If you answer more than 10 questions correctly, you will still get 200 yen.
  - If you answer fewer than 9 questions correctly, you will not receive any additional reward.

Attention

- You cannot return to the previous problem after moving on to the following problem.
- You have 5 minutes.
- Please write your target number. After that, please start the typing task.

Start of Actual Task

Fill out the receipt

- We will let you know the number of correct answers and the final amount for the typing task. You will then be asked to write down the final amount and sign in your own handwriting.

- If you are not satisfied with the number of correct answers, we will check the number of correct answers and compare them with your answers.
  - In such case, we will show you the answers after the entire experiment is over.

- No seal is required.

- The bank transfer will be made at the end of January or February.

Experiment

memo: This is the Info group.

(same) means the same as that in the Control group.

(difference) means the additional explanation unlike the Control group.

(same)

Please complete the following forms

- Please read the entire Economic Experiment Explanatory Document.

- Then, please fill out the following:
  - Economic Experiment Consent Form
  - Request for Bank Transfer Form

(same)

Economic Experiment Consent Form

- This section explains the important aspects of the Economic Experiment Consent Form.
- The experiment will be a typing task. It is scheduled to take 30 minutes, including payment.
- Your decisions and answers are guaranteed to be anonymous.
- If you are not satisfied with the specifics of the experiment, you can withdraw from participation at any time after reading the explanation. In such case, you will receive remuneration for your visit to the laboratory.
  - Payment is by bank transfer.
- If you have any questions, please ask the person in charge of the experiment.
- We will collect the Experiment Consent Form and Bank Transfer Request Form.

(same)

Payment of Participation Fee

- Thank you very much for your participation.
- The participation fee of 1,000 yen will be paid first.

Task

(same)

Rules of the show 1

- You will type the 12-digit number displayed on your monitor correctly.
- One problem is at 20 yen per correct answer.
- There will be a total of 75 typing problems (=20YEN ×75 problems=1500YEN (MAX)).
- You have 5 minutes.
- All problems consists of the 12-digit number (below)
- You can use the QWERTY keyboard or thenumeric keypad (10 keys) next to the QWERTY keyboards.

Exmples of typing problems

392832944950

958375930201

094726184959

(same)

Rule of the show 2

- You will be asked to set your target number of correct answers.
  - If you answer as many or more questions correctly than your target number of correct answers, you will be paid an amount equal to your target number of correct answers.
  - If you do not reach your target number of correct answers, you will only receive the participation fee.
- One problem is at 20 yen per correct answer.
- For example, let's say that your target number of correct answers is 10.
  - If you get more than 10 correct answers, you will get an additional 200 yen. If you answer more than 10 questions correctly, you will still get 200 yen.
  - If you answer fewer than 9 questions correctly, you will not receive any additional reward.

(difference)

- Here is the actual distribution of correct answers prior to the experiment.
- After confirmation, please push the "confirmation" button.

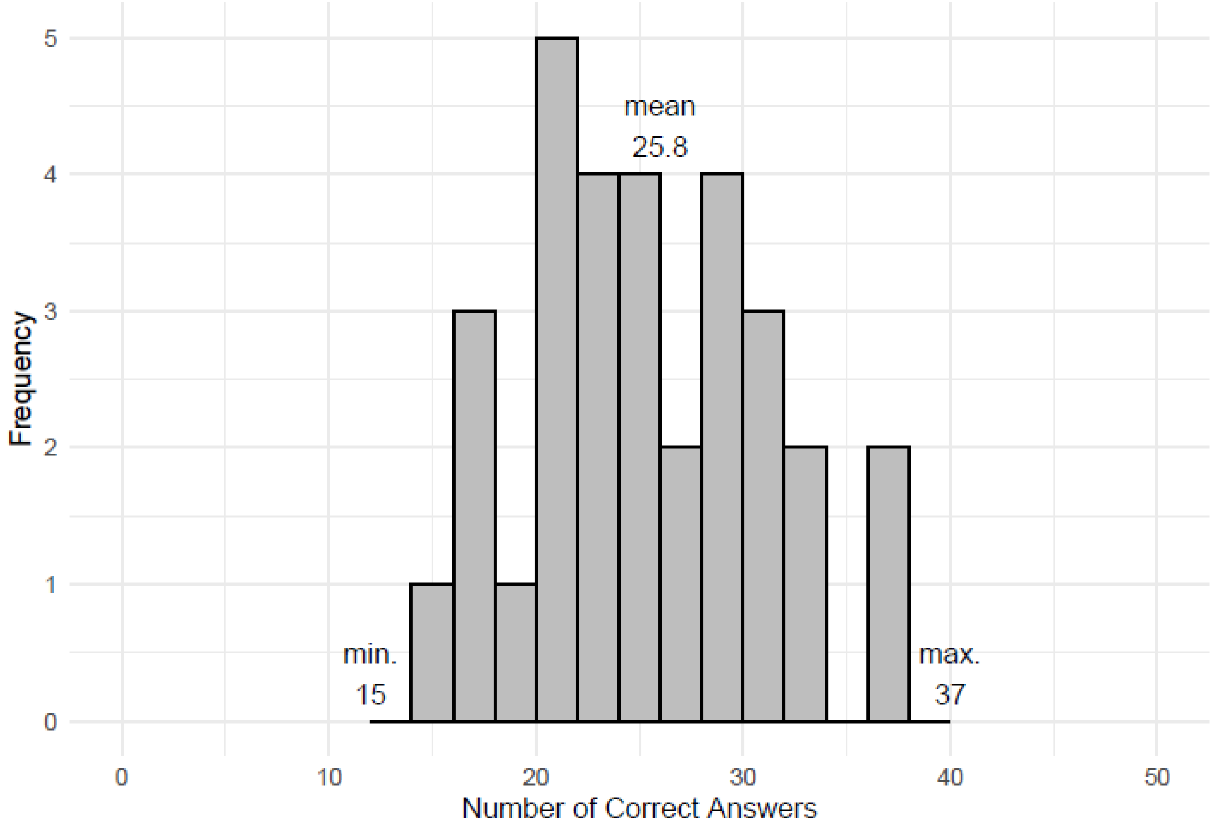

(same)

Attention

- You cannot return to the previous problem after moving on to the following problem.
- You have 5 minutes.
- Please write your target number. After that, please start the typing task.

(same)

Start of Actual Task

(same)

Fill out the receipt

- We will let you know the number of correct answers and the final amount for the typing task. You will then be asked to write down the final amount and sign in your own handwriting.

- If you are not satisfied with the number of correct answers, we will check the number of correct answers and compare them with your answers.
  - In such case, we will show you the answers after the entire experiment is over.

- No seal is required.

- The bank transfer will be made at the end of January or February.

Experiment

memo: This is the Exp group.

(same) means the same as that in the Control group.

(difference) means the additional explanation unlike the Control group.

(same)

Please complete the following forms

- Please read the entire Economic Experiment Explanatory Document.

- Then, please fill out the following:
  - Economic Experiment Consent Form
  - Request for Bank Transfer Form

(same)

Economic Experiment Consent Form

- This section explains the important aspects of the Economic Experiment Consent Form.
- The experiment will be a typing task. It is scheduled to take 30 minutes, including payment.
- Your decisions and answers are guaranteed to be anonymous.
- If you are not satisfied with the specifics of the experiment, you can withdraw from participation at any time after reading the explanation. In such case, you will receive remuneration for your visit to the laboratory.
  - Payment is by bank transfer.
- If you have any questions, please ask the person in charge of the experiment.
- We will collect the Experiment Consent Form and Bank Transfer Request Form.

(same)

Payment of Participation Fee

- Thank you very much for your participation.
- The participation fee of 1,000 yen will be paid first.

Task

(same)

Rules of the show 1

- You will type the 12-digit number displayed on your monitor correctly.
- One problem is at 20 yen per correct answer.
- There will be a total of 75 typing problems (=20 YEN ×75 problems=1500 YEN (MAX)).
- You have 5 minutes.
- All problems consists of the 12-digit number (below)
- You can use the QWERTY keyboard or thenumeric keypad (10 keys) next to the QWERTY keyboards.

Exmples of typing problems

392832944950

958375930201

094726184959

(same)

Rule of the show 2

- You will be asked to set your target number of correct answers.
  - If you answer as many or more questions correctly than your target number of correct answers, you will be paid an amount equal to your target number of correct answers.
  - If you do not reach your target number of correct answers, you will only receive the participation fee.
- One problem is at 20 yen per correct answer.
- For example, let's say that your target number of correct answers is 10.
  - If you get more than 10 correct answers, you will get an additional 200 yen. If you answer more than 10 questions correctly, you will still get 200 yen.
  - If you answer fewer than 9 questions correctly, you will not receive any additional reward.

(difference)

Practice task

(difference)

Practice task

- We have the practice task within 5 minutes.
- You cannot return to the previous problem after moving on to the following problem.
- You cannot earn anything in the practice task.
- After the practice task, you can see the number of your correct answer.
- The number of your correct answer in the practice task does not affect anything in the actual typing task.

(difference)

Actual task

(same)

Attention

- You cannot return to the previous problem after moving on to the following problem.
- You have 5 minutes.
- Please write your target number. After that, please start the typing task.

(same)

Start of Actual Task

(same)

Fill out the receipt

- We will let you know the number of correct answers and the final amount for the typing task. You will then be asked to write down the final amount and sign in your own handwriting.
- If you are not satisfied with the number of correct answers, we will check the number of correct answers and compare them with your answers.
  - In such case, we will show you the answers after the entire experiment is over.
- No seal is required.
- The bank transfer will be made at the end of January or February.

Experiment

memo: This is the InfoExp group.

(same) means the same as that in the Control group.

(difference) means the additional explanation unlike the Control group.

(same)

Please complete the following forms

- Please read the entire Economic Experiment Explanatory Document.
- Then, please fill out the following:
  - Economic Experiment Consent Form
  - Request for Bank Transfer Form

(same)

Economic Experiment Consent Form

- This section explains the important aspects of the Economic Experiment Consent Form.
- The experiment will be a typing task. It is scheduled to take 30 minutes, including payment.
- Your decisions and answers are guaranteed to be anonymous.
- If you are not satisfied with the specifics of the experiment, you can withdraw from participation at any time after reading the explanation. In such case, you will receive remuneration for your visit to the laboratory.
  - Payment is by bank transfer.
- If you have any questions, please ask the person in charge of the experiment.
- We will collect the Experiment Consent Form and Bank Transfer Request Form.

(same)

Payment of Participation Fee

- Thank you very much for your participation.
- The participation fee of 1,000 yen will be paid first.

Task

(same)

Rules of the show 1

- You will type the 12-digit number displayed on your monitor correctly.
- One problem is at 20 yen per correct answer.
- There will be a total of 75 typing problems (=20YEN ×75 problems=1500YEN (MAX)).
- You have 5 minutes.
- All problems consists of the 12-digit number (below)
- You can use the QWERTY keyboard or thenumeric keypad (10 keys) next to the QWERTY keyboards.

Exmples of typing problems

392832944950

958375930201

094726184959

(same)

Rule of the show 2

- You will be asked to set your target number of correct answers.
  - If you answer as many or more questions correctly than your target number of correct answers, you will be paid an amount equal to your target number of correct answers.
  - If you do not reach your target number of correct answers, you will only receive the participation fee.
- One problem is at 20 yen per correct answer.
- For example, let's say that your target number of correct answers is 10.
  - If you get more than 10 correct answers, you will get an additional 200 yen. If you answer more than 10 questions correctly, you will still get 200 yen.
  - If you answer fewer than 9 questions correctly, you will not receive any additional reward.

(difference)

- Here is the actual distribution of correct answers prior to the experiment.
- After confirmation, please push the "confirmation" button.

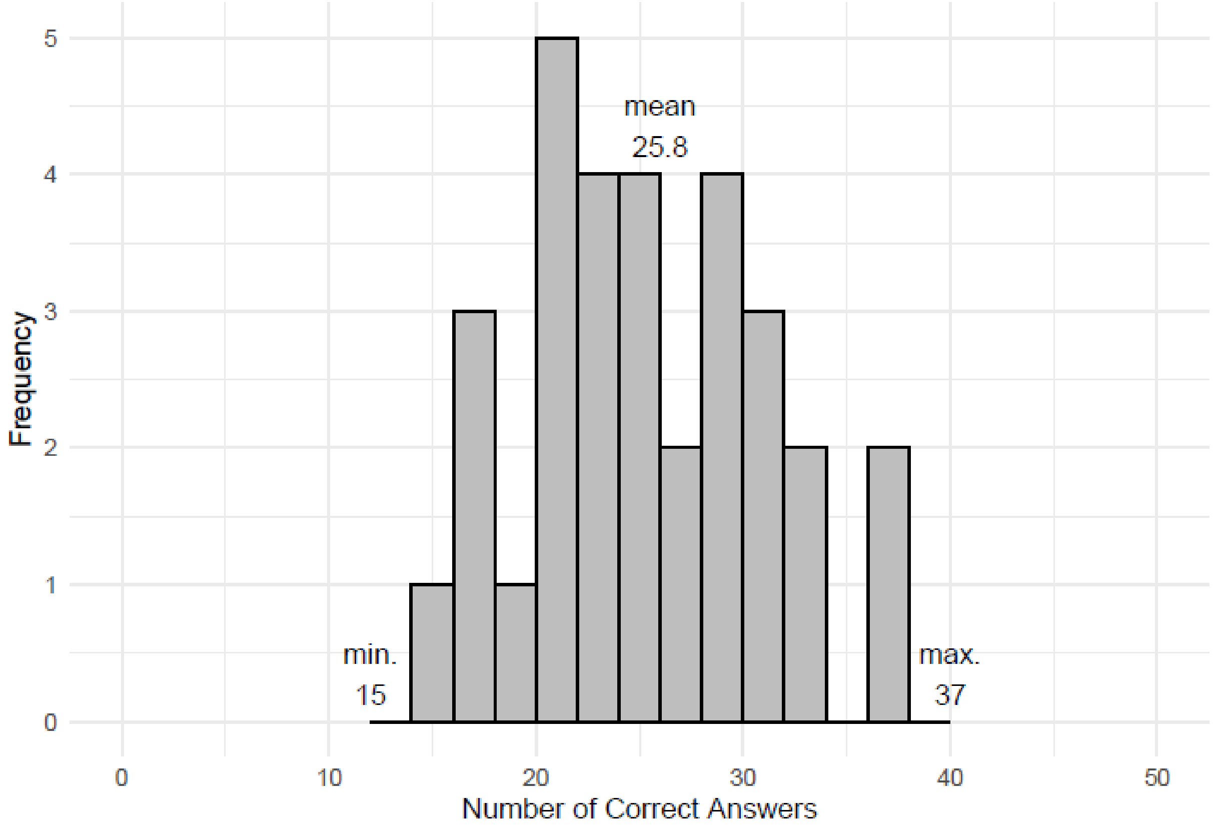

(difference)

Practice task

(difference)

Practice task

- We have the practice task within 5 minutes.
- You cannot return to the previous problem after moving on to the following problem.
- You cannot earn anything in the practice task.
- After the practice task, you can see the number of your correct answer.
- The number of your correct answer in the practice task does not affect anything in the actual typing task.

(difference)

Actual task

(same)

Attention

- You cannot return to the previous problem after moving on to the following problem.
- You have 5 minutes.
- Please write your target number. After that, please start the typing task.

(same)

Start of Actual Task

(same)

Fill out the receipt

- We will let you know the number of correct answers and the final amount for the typing task. You will then be asked to write down the final amount and sign in your own handwriting.
- If you are not satisfied with the number of correct answers, we will check the number of correct answers and compare them with your answers.
  - In such case, we will show you the answers after the entire experiment is over.
- No seal is required.
- The bank transfer will be made at the end of January or February.
